# Genotypic characterization of extended-spectrum β-lactamase producing urinary isolates among pregnant women in Ho municipality, Ghana

**DOI:** 10.1016/j.heliyon.2022.e12513

**Published:** 2022-12-22

**Authors:** Robert K. Adedze-Kpodo, Patrick K. Feglo, Eric Agboli, Richard H. Asmah, Precious K. Kwadzokpui

**Affiliations:** a South Tongu District Hospital, Sogakope, Ghana; b Department of Clinical Microbiology, School of Medical Sciences, Kwame Nkrumah University of Science and Technology, Kumasi, Ghana; c Department of Epidemiology and Biostatistics, School of Public Health, University of Health and Allied Sciences, Ho, Ghana; d College of Health Sciences, School of Biomedical and Allied Health Sciences, University of Ghana, Legon, Ghana; e Department of Medical Laboratory Science, School of Allied Health Sciences, University of Health and Allied Sciences, Ho, Ghana; f Medical Laboratory Department, Ho Teaching Hospital, Ho, Ghana

**Keywords:** Extended spectrum β-lactamase, Antimicrobial, Urinary bacteria, Pregnancy, Antenatal clinic

## Abstract

**Objective:**

The case of antibiotic resistance has become a major global concern and Extended Spectrum β-lactamase (ESBL) producing organisms have so far remained the biggest culprit. The consequences of urinary tract infection (UTI) and antibiotic resistance among pregnant women cannot be underestimated. We investigated UTI and ESBL production among urinary pathogens isolated from pregnant women.

**Method:**

We obtained non-repeat, clean catch midstream urine samples from 1345 pregnant women suspected of having UTI for bacterial identification at the Ho Teaching Hospital Laboratory between June 2013 and March 2015. The isolates were taken through relevant biochemical testing for identification and then subjected to antimicrobial agents for susceptibility testing using the disc diffusion method. We tested for ESBL production by the combined disc method and ESBL positive (+ESBL) phenotype isolates were genotyped for Bla_TEM_, Bla_SHV,_ and Bla_CTX-M_ using polymerase chain reaction (PCR). Data were analyzed using SPSS v24 and p-values < 0.05 were considered statistically significant.

**Results:**

Of the 1345 urine samples tested, 230 (17.1%, 95% CI: 15.1%–19.1%) yielded significant bacteriuria. The most common bacterium isolated was *Staphylococcus aureus* (29.6%) followed closely by *Escherichia coli* (28.7%) both of which were highest during the second trimester of gestation. We isolated 152 gram-negative isolates with 41.4% (63/230) being + ESBL. Of the 63 + ESBL, 45 (71.4%) possessed bla_TEM_, 42 (66.7%) had bla_CTX-M_ and 2 (3.2%) possessed bla_SHV_ genes; 38 possessed multiple ESBL genes comprising 2 with both SHV and TEM genes and 36 with both CTX-M and TEM genes.

**Conclusion:**

High prevalence of UTI and persistent transmission of ESBLs among pregnant women in the Ho Municipality is worrying and a course for public health concern. We recommend urine culture during pregnancy as a routine laboratory investigation to avoid birth-related complications.

## 1 Introduction

Worldwide, about 150 million people are diagnosed with UTI each year which may be uncomplicated or complicated [1]. The prevalence of UTI in Ghana was estimated as 56.5 % [2] higher than earlier reports of 9.5 % [3] and 3.3 % [4]. Bacteria-associated UTIs such as pyelonephritis are common during pregnancy causing significant maternal and neonatal complications, including threatened abortion, preterm labor, and neonatal mortality among others [5]. As a result of the high exposure to risk factors especially among pregnant women from rural communities of low-resourced settings, the administration of antibiotics to treat UTI during pregnancy has become a frequent practice. Unfortunately, aside from the extensive and sometimes irresponsible administration of beta-lactam antibiotics in clinical settings by clinicians without evidence of the exact pathogen responsible for the UTI, self-medication for the treatment of UTI is fast becoming a common practice during pregnancy, especially within the communities where education on the discriminatory use of medicines for various health conditions is on the low side. Additionally, non-compliance to administered antibiotics by patients, etc. has all contributed to the emergence and widespread dissemination of antibiotic-resistant bacteria resulting in a selective process for the bacterial strains that possess the resistance gene [6]. Prevalence of *Enterobacteriaceae* strains producing ESBLs varies widely internationally and as well as within the same country depending on the degree of use of the antibiotic in that locality [7, 8]. In Ghana, ESBL prevalence of 50.5% in Accra [9], 57.8% in Kumasi [10], and 41.5% at the Ho Teaching Hospital [11] were reported. Elsewhere, ESBL prevalence of 50% in northwestern Nigeria [12], 36.1% in South Africa (36.1%) [13], and 38.5% in Tunisia (38.5%) have been documented [14].

The introduction of second and third-generation cephalosporins into clinical use leads to enterobacteria developing ESBL enzymes to degrade them. ESBL enzymes are secreted to cleave to the beta-lactam ring of penicillin and cephalosporin [15] and thus provide resistance against these groups of antibiotics [16]. ESBL-producing bacteria are predominantly *Klebsiella pneumoniae* and *Escherichia coli* [17]. The predominant gene types they produce include SHV, TEM and CTX-M, OXA, PER, and VEB-1. The most common ESBL genes isolated from clinical specimens are bla_CTX-M_, bla_SHV,_ and bla_TEM_ [17]. It has been observed that the same organism may harbor two or more ESBL genes, which may change the antibiotic resistance phenotype [18] and hence render antibiotic therapy more difficult. The bla_CTX-M_ gene types are replacing bla_SHV_ and bla_TEM_ as the prevalent type of ESBLs in urinary tract infections, bloodstream and intra-abdominal infections [19]. The emergence of ESBL-producing pathogens has become increasingly significant in limiting antibiotic treatment options.

Urinary tract infection poses enormous challenges in pregnancy and remains one of the risk factors for morbidity and mortality. Urinary tract infections in pregnancy may lead to unfavorable pregnancy outcomes and complications like preterm delivery, low birth weight, pre-eclamptic toxemia, and anemia [20]. Most cases of pyelonephritis occur during the second and third trimesters, and complications include septic shock syndrome, anemia, bacteremia, respiratory insufficiency, and renal dysfunction [21]. There is a need to continually monitor and characterize the circulating ESBL-producing organisms, especially in vulnerable groups such as pregnant women. Community surveillance of ESBL-producing organisms remains one of the fundamental routes in profiling the extent of spread of the ESBL producers for relevant decision making. Because of the paucity of comprehensive data on ESBL-producing *Enterobacteriaceae* in Africa, specifically Ghana, and particularly among vulnerable populations such as pregnant women, this study determined ESBL occurrence and its genotypes circulating among pregnant women in the Ho Municipality.

## 2 Materials and methods

### 2.1 Study design and site

**Figure 1:**
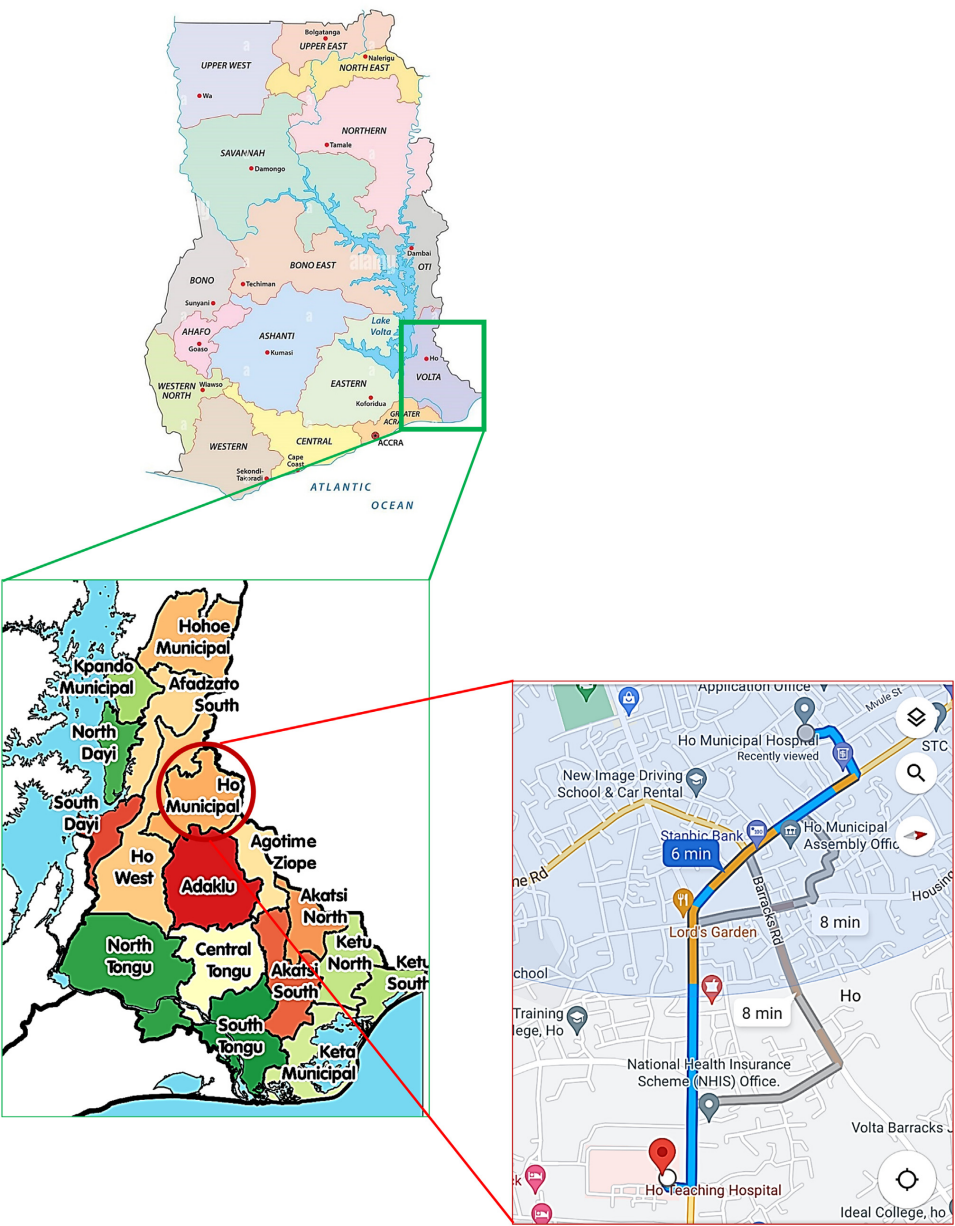
Map of Ho Municipal District showing the location of HTH and HMH. *Source*: Wikipedia, Google Maps.

This was a cross-sectional study conducted at the antenatal clinics of the Ho Municipal Hospital (HMH) and Volta Regional Hospital now the Ho Teaching Hospital (HTH) between June 2013 and March 2015. These two hospitals are located in the Ho Municipality. The distance between the two hospitals is about 2.5 km (Figure 1). The Ho Municipality which houses the two health facilities is situated between latitudes 6^o^ 36′ 43″ N and longitudes 0^o^ 28′ 13″ E. The Municipality shares boundaries with Adaklu and Agotime-Ziope Districts to the South, Ho West District to the North and West, and the Republic of Togo to the East. Its total land area is 2,361 square kilometers thus representing 11.5 percent of the region's total land area. The capital, Ho, is located about 158 km, from Accra, the National Capital. The major ethnic groups in the municipality are the Ewes, Guan people, and Akan folks. The population of Ho Municipality according to the 2010 Population and Housing Census is 177,281 representing 8.4 percent of the region's total population.

### 2.2 Study participants and eligibility criteria

This study recruited pregnant women who received antenatal services from the Ho Teaching Hospital and Ho Municipal Hospital. Pregnant women who were registered with the two hospitals but were not residents of the Ho Municipality were excluded from the study. This was to ensure that findings made from the study are accurate reflections of disease conditions among pregnant women from Ho Municipality only. We also excluded pregnant women who refused consent and participation in the study.

### 2.3 Data and specimen collection

A face-to-face interview was conducted using a structured questionnaire to collect sociodemographic data including maternal age and gestational period.

### 2.4 Urine sample collection and processing

About 10 mls of freshly voided midstream urine sample was collected into a pre-labeled (specimen collection date, time, identification code, and age) sterile, leak-proof, wide mouth, screw-capped plastic container (Viamed, Miami Lakes) by each pregnant woman following standard urine sample collection procedure. The urine samples were labeled appropriately and transported in an icebox to the Ho Teaching Hospital Laboratory and processed within 2 hours. No specimen required refrigeration since all specimens were processed within 2 h post-collection without delay.

### 2.5 Urine culture and identification of isolates

The urine samples were inoculated on Cystine Lactose Electrolyte Deficient age (CLED) (Oxoid Ltd, UK) medium using calibrated urine loop (1/1000ml) and incubated aerobically at 37 °C for 18–24 h (overnight culture). After overnight incubation, the plates were examined for growth, and the significance of growth was assessed by colony count. Bacterial colony counts of ≥1 × 10^5^ colony forming units per ml (CFU/mL) were considered significant bacteriuria and counts that fell between 10^2^–10^4^ CFU/mL were considered as suspected/doubtful bacteriuria while colony counts <10^2^ CFU/mL were considered not significant [22]. Cultures with significant bacteriuria were then followed-up with further testing for identification. Discrete colonies on CLED agar plates with significant growth were picked and inoculated onto nutrient agar and incubated at 37 °C for 18–24 h to obtain a pure culture. From the pure culture plate, the organisms were identified using the gram staining technique and various relevant biochemical tests. These included oxidase test to identify non-lactose fermenting bacteria, particularly *Pseudomonas spp*. Indole test for identification of enterobacteria such as *Proteus spp*., *E. coli* and also to differentiate *Proteus vulgaris* from *Proteus mirabilis*. Urease test to differentiate *Proteus spp*. from other enterobacteria. The coagulase test was used to differentiate *Staphylococcus aureus* from coagulase negative *Staphylococci.* Novobiocin was used to differentiate between *Staphylococcus saprophyticus* from *Staphylococcus epidermidis.* Triple Sugar Iron (TSI) agar test was also used to differentiate between the various enterobacteria [22]. All follow-up biochemical testings were done following standard clinical laboratory procedures [22].

### 2.6 Extended-spectrum beta-lactamase detection (screening and confirmation)

All gram-negative isolates were screened for the presumptive presence of ESBLs. Isolates with reduced susceptibilities to cefotaxime (30 μg) (zone diameter of <27 mm) and/or ceftazidime (30μg) (zone diameter of <22 mm) were suspected to be +ESBL in the screening test [23]. ESBL phenotypic detection among the isolates was then confirmed using the combined disc method (OxoidHamshire, England) according to the Clinical and Laboratory Standards Institute [23]. The test was performed by spreading the organism to be tested on Mueller Hinton agar then after, ceftazidime (30 μg) disk and cefotaxime (30μg) disk were used alone and then in combination with 10 μg clavulanic acid (Becton Dickinson) and then incubated at 37 °C for 18–24 h for phenotypic confirmation of the presence of +ESBL. Isolates were considered + ESBL if zone diameters increased by ≥ 5 mm for either cefotaxime or ceftazidime alone and with the corresponding antibiotic in combination with clavulanic acid [23].

### 2.7 Extended-spectrum beta-lactamase genotyping

All confirmed + ESBL gram-negative isolates were further tested for ESBL genes. The genes tested for were bla_TEM_*,* bla_CTX-M,_ and bla_SHV_. DNA was extracted by the method of boiling [24]. PCR was carried out using BIOER Gene Pro thermocycler. Total reaction mixture of 25μl for a primer set was prepared. These include Nuclease free water (14.574μl), 10X PCR buffer + MgCl_2_ (2.5μl), 10mM dATP (0.5μl), 10mM dCTP (0.5μl), 10mM dGTP (0.5μl), 10mM dTTP (0.5μl), 10μM forward primer (0.4μl), 10μM reverse primer (0.4 μl), 5Uμl Taq polymerase (0.125μl), and DNA Template (5μl) for the detection of bla_TEM_*,* bla_CTX-M_ and bla_SHV_ (Table 1).

**Table 1:** Nucleotide sequences of PCR primers used to amplify the ESBL genes.

Primer name	Sequence (5′-3′)	Target gene	Amplicon Size (bp)
**TEM- F**	5′-TCCGCTCATGAGACAATAACC-3′	**TEM**	**1058**
**TEM -R**	5′-TTGGTCTGACAGTTACCAATGC-3′		
**SHV- F**	5′-TGGTTATGCGTTATATTCGCC-3′	**SHV**	**865**
**SHV- R**	5′-GGTTAGCGTTGCCAGTGCT-3′		
**CTX-M-F**	5′-TCTTCCAGAATAAGGAATCCC-3′	**CTX-M**	**910**
**CTX-M-R**	5′-CCGTTTCCGCTATTACAAAC-3′		

### 2.8 PCR conditions used for the detection of ESBL genes

The PCR conditions used for the detection of ESBL genes vary. The target gene CTX-M used initial denaturation for 5 min at 94 °C; 25 cycles of 94 °C for 30s, 52 °C for 30s, 72 °C for 60 s; final extension at 72 °C for 7 min. The target gene TEM used initial denaturation for 5 min at 94 °C; 30 cycles of 94 °C for 30 s, 52 °C for 30 s, 72 °C for 60 s; final extension at 72 °C for 7 min. Initial denaturation for 5 min at 95 °C; 30 cycles of 95 °C for 60 s, 55 °C for 60 s, 72 °C for 60 s; final extension at 72 °C for 10 min were used for SHV target gene.

### 2.9 Agarose gel electrophoresis

A 1XTAE buffer was prepared and used to prepare 2% agarose gel. The suspension was melted in a microwave for 2 min. The molten agarose was stained with 3 μl of 0.5 μg/ml ethidium bromide. The molten agarose was poured into the tray with a comb inserted and allowed to set and solidify. Two microliters (2 μl) of 10× Orange G loading dye was added to 10 μl of each PCR product and loaded into the wells. The 100 bp DNA ladder was loaded as a marker and the gel was electrophoresed at 120 V for 45 min. The bands on the gels were visualized by ultraviolet trans-illumination and photographed by Kodak camera.

### 2.10 Quality assurance

All necessary quality control checks were instituted before, during and after data collection. Data collection was done only by trained data collectors via face-to-face interviews in a language best understood by the participants (thus English language, Eve and Twi). Data completeness and consistency checks was performed after which a fit-for-purpose Microsoft Excel 2016 was used to capture the data to avoid as many as possible data entry errors. All quality control checks of laboratory assays and media were checked. This included inspection for expiration date and after media preparation, visual inspection for cracks in media due to dryness of media, rough media surfaces due to bubbles, unequal fill of the media plates and evidence of contamination. *Escherichia coli* ATCC 25922 was used as the ESBL negative (−ESBL) control strain while *Klebsiella pneumoniae* ATCC 700603 was used as the ESBL positive (+ESBL) control strain.

### 2.11 Research questions

1. What is the prevalence of UTI among pregnant women in the Ho Municipality?
2. What is the prevalence of ESBL producers and what are the ESBL genes and distribution among pregnant women in the Ho Municipality?

### 2.12 Ethical approval and consent to participate

Approval for the study was obtained from the Joint Committee on Human Research, Publication and Ethics (CHRPE) of Komfo Anokye Teaching Hospital (KATH)/School of Medical Sciences (SMS) (Ref: CHRPE/RC/041/13). Permission was obtained from Ho Teaching Hospital and Ho Municipal Hospital administration for the conduct of this study. All participants signed an informed consent form after thorough explanation of the study to them. All specimens were labeled using a unique coding system for each participant's urine sample without taking any personally identifiable details to ensure confidentiality.

### 2.13 Statistical analysis

Data collected we recorded into Microsoft excel 2016 using fit-for-purpose excel forms to minimize entry errors. Captured data were then checked for consistency and then transferred into IBM SPSS statistical software version 24 for statistical analysis. Data are presented as frequencies and percentages in tables. The Chi-square test statistic was used to determine the association between independent and outcome variables of interest. P-values of less than 0.05 were considered statistically significant.

## 3 Results

### 3.1 Urine culture

This study recruited pregnant women aged 15–45 years with the majority and the least falling within the age category of 25–29 years old (27.7%) and 35–45 years old (10.4%) respectively. Out of the 1345 urine samples cultured, 230 had significant bacteria growth giving a UTI prevalence of 17.1% (95% CI: 15.1%–19.1%) among pregnant women. The highest UTI prevalence was recorded among pregnant women aged 25–39 years (19.6%) while the least was recorded among those aged 15–19 years (13.0%).

### 3.2 Prevalence of urinary tract isolates stratified by gestational age

Findings from this study revealed a higher prevalence of UTI among pregnant women in the second trimester (42.6%). The most predominating organism isolated was *S. aureus* (29.6%) followed closely by *E. coli* (28.7%) both of which were highest among pregnant women in their second trimester of gestation (Table 2).

**Table 2:** Prevalence of urinary tract isolates stratified by gestational age.

Isolates	Trimester			
Overall	First (%)	Second (%)	Third (%)	Overall (%)
	45 (19.6)	98 (42.6)	87 (37.8)	230 (17.1)
*Staphylococcus aureus*	11 (16.2)	30 (44.1)	27 (39.7)	**68 (29.6)**
*Escherichia coli*	12 (18.2)	29 (43.9)	25 (37.9)	**66 (28.7)**
*Citrobacter spp*	10 (35.7)	9 (32.1)	9 (32.1)	**28 (12.2)**
*Klebsiella pneumoniae*	1 (5.0)	14 (70.0)	5 (25.0)	**20 (8.7)**
*Pseudomonas aeruginosa*	3 (16.7)	7 (38.9)	8 (11.1)	**18 (7.8)**
*Enterobacter spp*	3 (21.4)	6 (42.9	5 (35.7)	**14 (6.1)**
*Staphylococcus saprophyticus*	4 (40)	2 (20.0)	4 (40.0)	**10 (4.3)**
*Providentia spp*	1 (8.1)	1 (20.0)	3 (60.0)	**5 (2.2)**
*Proteus mirabilis*	0 (0.0)	0 (0.0)	1 (100.0)	**1 (0.4)**

**Figure 2:**
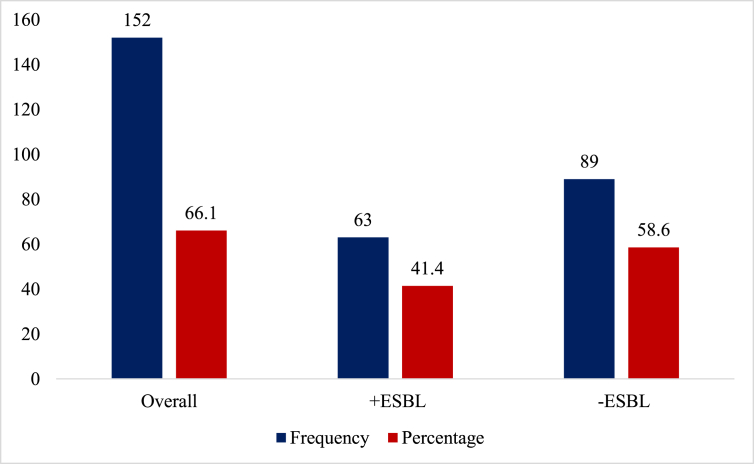
Prevalence of urinary-associated ESBL producers among pregnant women.

As shown in Figure 2 below, of the overall 152 significant bacteriuria, nearly half, 41.4% were +ESBL whereas 58.6% were -ESBL.

### 3.3 Extended-spectrum beta-lactamase gene types from gram negative isolates among pregnant women

Of the 63 + ESBL, 45 (71.4%) possessed TEM genes with a bands amplicon size of 1058 bp, 42 (66.7%) had CTX-M genes with a bands amplicon size of 910 bp and 2 (3.2%) had SHV genes with a bands amplicon size of 865 bp (Table 3).

**Table 3:** Prevalence of +ESBLs and the associated ESBL genotypes.

Isolates	+ESBL Isolate (%)	ESBL Genotype		
		TEM (%)	CTX-M (%)	SHV (%)
Total (%)	63 (41.4)	45 (71.4)	42 (66.7)	2 (3.2)
*Escherichia coli*	23 (34.8)	9 (39.1)	4 (17.4)	2 (8.7)
*Klebsiella pneumonia*	13 (65.0)	13 (100)	13 (100)	0 (0.0)
*Citrobacter spp*	11 (39.3)	7 (63.6)	9 (81.8)	0 (0.0)
*Pseudomonas aeruginosa*	8 (44.4)	8 (100)	8 (100)	0 (0.0)
*Enterobacter spp*	6 (42.9)	6 (100)	6 (100)	0 (0.0)
*Providentia spp*	2 (40.0)	2 (100)	2 (100)	0 (0.0)

The prevalence of gram-negative bacteriuria was higher (43.4%, 66/152) in the second trimester of gestation. Further analysis revealed the highest + ESBL among pregnant women was recorded in their second trimester (45.5%) whereas −ESBL was highest among pregnant women in their third trimester (62.5%). Meanwhile. Gestational age was not significantly associated with ESBL production (Table 4).

**Table 4:** Prevalence of ESBL production stratified by gestational age.

Gestational Age	Total GNI	+ESBL	−ESBL
1st Trimester	30 (19.7)	12 (40.0)	18 (60.0)
2nd Trimester	66 (43.4)	30 (45.5)	36 (54.5)
3rd Trimester	56 (36.8)	21 (37.5)	35 (62.5)
Chi-square (p-valued)		0.82 (0.6629)	

GNI-Gram-negative isolates; +ESBL- Extended Spectrum Β-lactamase producers; −ESBL- Extended Spectrum Β-lactamase nonproducers.

In this study, of the 63 + ESBL isolates, 38 possessed multiple ESBL genes. Of the 38, 2 had SHV and TEM genes whereas 36 had CTX-M and TEM genes. None of the isolates possessed all three ESBL genes (Table 5).

**Table 5:** Frequency of multiple ESBL genes.

Two genes			Three genes
SHV	SHV	CTX-M	SHV + CTX-M + TEM
+	+	+	
CTX-M	TEM	TEM	
0	2	36	0

## 4 Discussion

The study reports on urinary tract bacterial pathogens and ESBL production among isolates obtained from pregnant women in Ho Municipality. We found an overall UTI prevalence rate of 17.1% (15.1%–19.1%) which was higher among pregnant women aged 25–39 years (19.6%) and in their second trimester (42.6%). The most predominating organism isolated was *S. aureus* (29.6%) followed closely by *E. coli* (28.7%) both of which were highest among pregnant women in their second trimester of gestation. The overall prevalence of +ESBL was 41.4% (Figure 2) which was higher among pregnant women in their second trimester. Finally, we found that 71.4%, 66.7%, and 3.2% of the +ESBLs possessed TEM, CTX-M, and SHV genes respectively whereas 2 had both SHV and TEM and 36 had both CTX-M and TEM genes.

The overall prevalence of UTI among pregnant women as observed in this study was comparable to the 16% among women during pregnancy in Sudan [25]. The UTI prevalence recorded in this study is however higher than earlier reports of 9.5 % in Kumasi, Ghana [3] and 12% in Saudi Arabia [26] but lower than 56.5% among pregnant women in Cape Coast, Ghana [2]. The variation in UTI prevalence from one geographical location to another could be attributed to differences in UTI perception, mode of screening, and risk factors such as age and parity [3]. Of note is the high UTI prevalence among pregnant women aged 25–39 years old. This however does not come as a surprise as this age group represents the most reproductive and sexually active age group. The vaginal organisms gain access to the urethra during sexual intercourse [27], leading to an increased tendency of bacteria to be massaged into the urethra with their subsequent migration into the bladder to initiate infection. Meanwhile, the natural close proximity of the female urethra to the anus makes it prone to fecal flora contamination, especially for pregnant women who fail to exhibit personal hygiene during gestation. The high UTI prevalence within this age group raises serious concerns about negative birth outcomes as a function of the infection which treatment may be challenged in the era of significant antibiotic resistance.

Generally, higher UTI prevalence was observed among pregnant women in their second and third trimesters compared to the first trimester. This observation could be accounted for by the anatomical and physiological changes which occur during pregnancy [28]. For example, the expansion of the uterus in pregnancy coupled with increased hormonal effects offsets normal homeostatic balance creating conditions favorable for microbial invasion [29]. Nonetheless, though UTI prevalence in the first trimester was relatively low, it served as an incubation period for most of the microbes, hence, UTI could not be diagnosed with significant bacterial counts at this stage. Besides the pregnant woman might have competent immune systems to keep the bacterial growth in check in the first trimester, and so as the pregnancy grows the women develop lower immune status and the UTI is manifested in the second and third trimesters [30].

The most prevalent organism observed in this study was *Staphylococcus aureus* followed closely by *Escherichia coli*. The prevalence of *Staphylococcus aureus* (29.6%) observed in this study was comparable to 27.1% in Nigeria in 2007 [31]. *Staphylococcus aureus* has been reported as the most prevalent UTI organism amongst pregnant women in Nigeria [31, 32] but in Tanzania [33] and Ghana [34], *Escherichia coli* was reported as the most prevalent organism isolated from urine. Both organisms; *E. coli* and *S*. *aureus* are believed to cause cystitis in many young sexually active females [35]. Variations in their prevalence among pregnant women within countries could be due to the general prevalence of the organism within the country's population as well as the personal hygiene practices of the studied population. The high *Staphylococcus aureus* prevalence recorded in this study could be community-acquired which may pose serious health challenges to the safety of pregnant women in the Ho Municipality.

In this study, 41.4% + ESBL bacteria were recorded. Other studies in Ghana reported 50.5% in Accra [9] and 57.8% in Kumasi [10]. Elsewhere, ESBL prevalence of 50% from northwestern Nigeria [12], 42% in Lagos, Nigeria [36], South Africa (36.1%) [13], Tunisia (38.5%) [14], and Tanzania (15%) [33] was reported. The ESBL genotypes obtained in Ho Municipality among the pregnant women were similar to those obtained in Accra [9] and a much earlier study that recorded an ESBL prevalence of 41.5% at the Ho Teaching Hospital [11]. In the present study, the prevalence of Bla_TEM_ was 45 (71.4%), Bla_CTX-M_ 42 (66.7%), and Bla_SHV_ 2 (3.2%). Clearly, the ESBL genotype is circulating within the communities and is largely affected by the degree of antibiotic usage. The ESBLs are produced in hospitals where the use of antibiotics is very high and the ESBL spreads to the communities. This might explain why a study conducted in Kumasi found high Bla_TEM_ and Bla_CTX-M_ (96.2% and 94.4%, respectively) genotypes [10]. The differences might be due to the inclusion of hospitalized patients in the Kumasi study compared to our study which comprised only patients from the community. This means that there is high prevalence of community-acquired ESBL circulating in the Ho Municipality. The high ESBL prevalence observed in this study holds the potential to result in significant treatment failure and the narrowing of the antibiotic spectrum due to its destructive effect on cephalosporins [11]. Meanwhile, when we consider the growing level of self-medication in our communities without prior laboratory investigation coupled with the difficulty in detecting ESBLs and the inconsistencies in reporting ESBLs within the community, we fear a surge in antibiotic resistance moving forward and the consequences among pregnant women may be dire.

## 5 Conclusions

The prevalence of UTI was 17.1% among pregnant women attending antenatal clinics in Ho Municipality. There was a high prevalence (41.4%) of +ESBL organisms among the isolates which were community-acquired isolates, with the *Bla*_TEM_ and *Bla*_CTX-M_ being the commonest.

## Declarations

### Author contribution statement

Robert K. Adedze-Kpodo: Conceived and designed the experiments; Performed the experiments; Analyzed and interpreted the data; Contributed reagents, materials, analysis tools or data; Wrote the paper.

Patrick K. Feglo: Performed the experiments; Contributed reagents, materials, analysis tools or data; Wrote the paper.

Eric Agboli; Richard H. Asmah: Performed the experiments; Contributed reagents, materials, analysis tools or data.

Precious K. Kwadzokpui: Performed the experiments; Analyzed and interpreted the data; Wrote the paper.

### Funding statement

This research did not receive any specific grant from funding agencies in the public, commercial, or not-for-profit sectors.

### Data availability statement

Data will be made available on request.

### Declaration of interests statement

The authors declare no competing interests.

### Additional information

Supplementary content related to this article has been published online at [URL].

## Appendix A Supplementary data

Questionnaire Questionnaire
