# Supplementary material for: Genotypic characterization of extended-spectrum β-lactamase producing urinary isolates among pregnant women in Ho municipality, Ghana
Source: Heliyon. 2022 Dec 22;8(12):e12513. doi: 10.1016/j.heliyon.2022.e12513 (PMC9803828; doi:10.1016/j.heliyon.2022.e12513)
Supplement: Questionnaire [file mmc1.docx]

**QUESTIONNAIRE**

**Identification Number…………………… Date …../…../20…..**

**Consent has been read to participant** Yes [ ] No [ ]

**1.0 DEMOGRAPHIC INFORMATION**

**1.1 Age [** ] years

**1.2 Trimester of pregnancy** [ ]

**2.0 CLINICAL HISTORY/INFORMATION**

**2.1 Is subject diagnosed of any clinical condition?** Yes [ ] No [ ]

If **yes,** indicate whether subject has any of the following conditions (to be marked if present)

2.2.1 Chronic and recurrent pelvic inflammatory disease. 2.2.2 Haematuria.

2.2.3 Renal disease. 2.2.4 Vaginal discharge.

2.2.5 Risk factor or diagnosed complication of pregnancy. 2.2.6 Ectopic pregnancy.

2.2.7 Bleeding after sexual intercourse. 2.2.8 Lumbar pain.
